# Head-Mounted Miniature Motorized Camera and Laser Pointer Driven by Eye Movements

**DOI:** 10.3390/s23073503

**Published:** 2023-03-27

**Authors:** Vincent Nourrit, Jean-Baptiste Lamour, Bernard Abiven, Bruno Fracasso, Jean-Louis de Bougrenet de la Tocnaye

**Affiliations:** 1 Optics Department, IMT Atlantique, 29238 Brest CEDEX 03, France; vincent.nourrit@imt-atlantique.fr (V.N.);; 2 Eyes Triple Shut, 78430 Louveciennes, France; 3 LaTIM, INSERM UMR1101, 22 Avenue Camille Desmoulins, 29200 Brest, France

**Keywords:** eye tracking, pan-tilt, point-of-view shot, eye movements, piezo-actuator, wearable camera, Risley prism, diffractive optical element

## Abstract

Recording a video scene as seen by an observer, materializing where is focused his visual attention and allowing an external person to point at a given object in this scene, could be beneficial for various applications such as medical education or remote training. Such a versatile device, although tested at the experimental laboratory demonstrator stage, has never been integrated in a compact and portable way in a real environment. In this context, we built a low-cost, light-weight, head-mounted device integrating a miniature camera and a laser pointer that can be remotely controlled or servo-controlled by an eye tracker. Two motorizations were implemented and tested (pan/tilt and Rilsey-prisms-based). The video was both recorded locally and transmitted wirelessly. Risley prisms allowed finer remote control of camera or laser pointer orientation (0.1° vs. 0.35°), but data processing and Wi-Fi transmission incur significant latency (~0.5 s) limiting the servo-controlling by eye movements. The laser beam was spatially shaped by a Diffractive Optical Element to facilitate object illumination or recognition. With this first proof-of-concept prototype, the data stream needs to be optimized to make full use of the eye tracker, but this versatile device can find various applications in education, healthcare or research.

## 1. Introduction

In past decades, a wide range of head-mounted devices has been developed to record what people are looking at, ranging from bulky prototypes [1] to modern action cameras [2]. Such systems find applications in various domains, such as entertainment (cinema, sport [3]) or psychological studies [4]. They can also be useful for subjective documentation in numerous professions such as, e.g., education, tele-maintenance, marketing, surveillance, etc. In the context of surgery in particular, it could be used to document the intervention, to better educate students by showing them the true point of view (POV) of a skilled medical professional, to compensate for decreased operative exposure [5,6] or after surgery to review the act and look for possible improvements [7]. It would also be useful for tele-assistance, helping a remote surgeon to supervise the surgery, and could be part of the operating room (OR) black-box efforts to reduce adverse events in the OR [8,9], particularly those related to human deficiencies [10].

Unfortunately, current solutions present several limitations. Solutions integrating a fixed camera, usually in the overhead lighting system, do not allow recording of the precise surgical gestures and the surgeons may position themselves in front of the camera. A head-mounted camera bypasses this problem but leads to other issues. Firstly, head-mounted cameras, usually action cameras, are rarely suited for use in a surgical theater. In addition to possible issues related to the weight distribution of the device or the heat generated, these cameras usually use a wide-angle lens that offers poor resolution and may not offer a satisfying scene framing, e.g., if it slips around its balance position or if the user looks at the periphery of the recorded frame. Even if the field of view can be adjusted, it has to be performed by the wearer, which is hardly practical during surgery. Last but not least, the video recording usually appears jerky and unpleasant to review due to random head shaking and/or spinning. Current head-mounted eye trackers with a forward-facing “world” camera that allows recording of the scene in front of the user suffer from the same limitations, but present an added value as they allow highlighting of the gaze direction in the associated video, and, in addition, to provide important behavioral information such as fatigue or cognitive load [11,12].

In this context, a way to improve the relevance of the recording and interactions with a distant expert would be to motorize the camera and to add a motorized laser pointer. The remote expert could use the motorized laser pointer to point to any element of interest and orientate the motorized camera to focus on the relevant elements. Alternatively, the scene camera or pointer could also be servo-controlled by the gaze direction for a better POV, to materialize the gaze direction and possibly to help improve image stabilization. In the 2000s, Brandt’s group built a sophisticated gaze-driven camera [13]. The system combined a wide-angle, head-fixed scene camera and a camera servo-controlled by a custom eye tracker. The authors aimed at a highly performant device: the camera orientation was to be stabilized only by the human vestibulo-ocular reflex. This camera could thus move along the vertical and horizontal axes (pan/tilt) as well as the line of sight (roll), thanks to a complex mechanical system involving piezo-actuators, push rods, universal joints and bearings [14]. The high degree of sophistication, together with available technologies at that time, led to a relatively bulky system.

Based on these observations, the aim of this work was to build and test a low-cost head-mounted device integrating a motorized camera and a laser pointer, that could be controlled remotely, or servo-controlled by a commercial eye tracker. The design philosophy of the device took advantage of advances in (i) high-performance additive mechanical design and (ii) integrated actuation and sensor systems driven by microcontrollers with an extremely desirable price/size/performance ratio. In this study, we initially chose to use off-the-shelf and compact components to reduce costs and facilitate prototyping and integration. Using additive manufacturing (3D printing) design methods allows optical, electronic and mechanical modules to be positioned and interfaced with great flexibility in small volumes, using lightweight materials and at moderate cost. In a second step, we propose a new beam-steering function to improve angular accuracy and control. The different components used are described in Section 2. Results on tracking, integration and performances are then presented (Section 3), followed by a discussion on the potential of the device.

## 2. Materials and Methods

As previously explained, the aim of this work was to build and test a low-cost head-mounted device integrating a motorized camera and a laser pointer, that could be controlled remotely or servo-controlled by a commercial eye tracker. The different elements that make up this device and the reasons for choosing them according to their specifications are presented hereafter, as well the design and integration procedure.

### 2.1. Eye Tracking

The eye-tracker used was the Pupil Labs’ Core model, which offers binocular tracking at a 200 Hz framerate. This model was chosen for its performance (0.6° angular accuracy), light weight (~23 g), affordability, and above all its open source software that allows for controlling the eye tracker through simple Python instructions. Basically, in video-based eye trackers such as the Pupil Labs’, one or multiple camera(s) take an image of the eyes, which are often illuminated by infrared light sources. Gaze direction is deducted from analysis of pupil and corneal reflections positions [15].

### 2.2. Motorization

**Figure 1:**
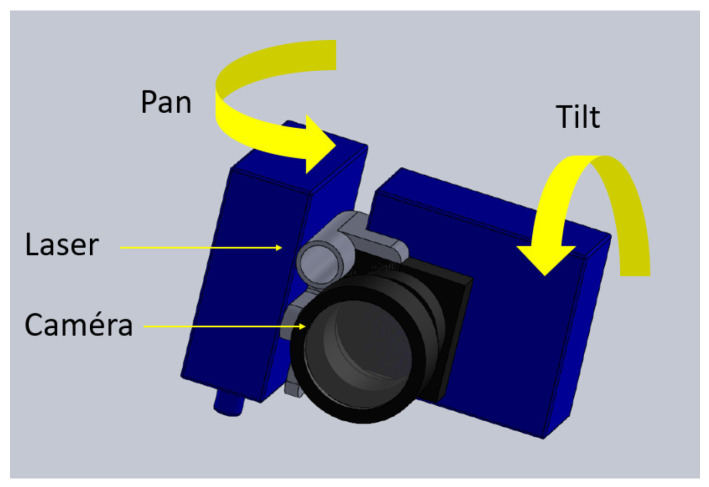
Pan-tilt motorization of the camera and laser pointer based on the combination of two servo-motors.

Motorization is a key function of the device. We initially chose to use a miniature pan-tilt setup because it allows directing the camera or pointer freely in any direction over almost 2π steradians field-of-view, and can be achieved using basic servo-motors. Two types of servo-motors were tested. The first motorization is based on two Hitec HS-40 servomotors. The plastic devices are small in size (20 × 8.6 × 17 mm each) and very lightweight (4.8 g). When driven at 4.8 V, the torque (0.6 N·m) allows moving the camera at 0.09 s/60°, which is theoretically fast enough to follow most eye movements. The coupled arrangement of the servo-motors holding the miniature camera is presented in Figure 1. To further reduce the weight and size, another motorization based on two D1302 nano-servo-motors was also tested (20.1 × 6.2 × 13.5 mm, 1.7 g; speed 0.06 s/60 °). However, at this level of compactness, there is a reduced mechanical torque (0.2 N·m) which penalizes the reactivity of the system.

The small size and the weight of the components make it possible to consider the integration of two motorizations, one for the camera, the other for the pointer. However, since the aim of our study was to demonstrate a proof of concept, we chose, for simplicity, to place both the camera and laser pointer on the same motorization.

To ensure that the camera or pointer is aimed where the user is looking, the following method was used, which basically consists of making the reference frame of the eye tracker and that of the pan-tilt motorization coincide. The eye tracker provides gaze data in its world camera’s field of view. The amplitude of movement of the pan-tilt was therefore limited to stay within this field. Then the origin of the pan-tilt system was adjusted so that the laser spot was located at the center of the image returned by the world camera (so that the origins of both reference frames correspond). From there, the gaze position returned by the eye tracker can be easily converted into two angles to be sent to the pan-tilt motors to adjust its orientation. Basically, the Pupil Labs returns the x, y gaze position in the world image frame in normalized coordinates. Since the angular size of this image is known, calculation of the angular gaze position is straightforward. The eye tracker was then calibrated using Pupil Labs software. The good agreement between the laser spot and fixation target was assessed during this calibration. This approach can, of course, lead to a bias of a couple of degrees, but this will have a limited impact, especially in the case of a wide-field camera.

### 2.3. Cameras

Two cameras were integrated into the headband: the eye tracker’ scene camera that provides a large field of view (103° × 54°) and the motorized one (150° × 60°). The motorized camera (OV2640) is a widely used model in the field of aerial drones. It offered a good compromise between performances, size of (9 × 9 mm), weight (5 g) and connectivity to the ESP32-CAM micro-controller. The camera can record high-definition images (1600 × 1200 pixels) at 15 fps, but we preferred to use it in low resolution mode (440 × 440) at maximum speed (50 fps), since the eye tracker operates at up to 200 Hz. The main difficulty when using off-the-shelf components is the control card that comes with the camera, which often takes up much more space than the camera itself, and the form factor makes it difficult to integrate it into a headband. However, we have managed to find integration solutions to meet this constraint.

### 2.4. Optical Sources

Two light sources were integrated into the headband. The first one is a LED XLamp^®^ XM-L (8 g) with an optic to have a homogeneous luminous flux, that facilitates image recording and helps the user with additional lighting directed on the area of interest. The second is a miniature laser source that can be combined with a diffractive optical element (DOE). The role of the DOE is to spatially shape the beam emitted by the laser into a prescribed structured pattern. This can be very useful to designate one or more objects in the scene (video analysis) or to help understand the spatial arrangement of objects in the scene being analyzed (e.g., projecting markers that would serve as control points for subsequent image registration). Two compact laser sources have been tested: a fiber laser emitting 5 mW and a laser diode with reduced power (2 mW; 6 × 0.5 × 0.5 cm; 10 g). Both lasers emit light at 650 nm (alternative wavelengths could be chosen) and produce a spot 3 mm large at 80 cm. They are collimated to focus the light on the areas of interest in the scene, in accordance with the camera’s sensitivity settings. The advantages of a fiber laser are twofold. Firstly, to move the source electronics out of the headband, and secondly, to easily change the laser source, by a simple external connection to the fiber.

The wavelength was chosen to facilitate testing and because there exists a wide choice of small low-cost red lasers. Also, our objective was to be able to point out for the user a particular object or area of interest, so the laser had to be visible. In this scenario, since the laser can be switched off remotely (via the ESP32), the fact that the laser is visible is thus not an issue. If the laser was constantly turned on, e.g., to monitor the gaze position, then it would be advisable to use an infrared laser (e.g., 850 nm) together with an IR camera so as not to disturb the user.

The DOEs tested were designed to produce structured illumination at 80 cm, which would correspond to a working distance for a surgeon. Three patterns were tested: a cross-hair shape, a target (concentric circles) and a dot matrix, but other illumination patterns (symbols or shapes) could be produced to facilitate image post-processing (e.g., registration, organ illumination, etc.). The DOE pattern was calculated using standard iterative Fourier-transform algorithm techniques [16]. It was then manufactured using a photolithography technique [17] on a glass plate and cut to a size of 4 mm × 4 mm. Diffraction pattern results will be shown in Section 3.

### 2.5. System Integration

**Figure 2:**
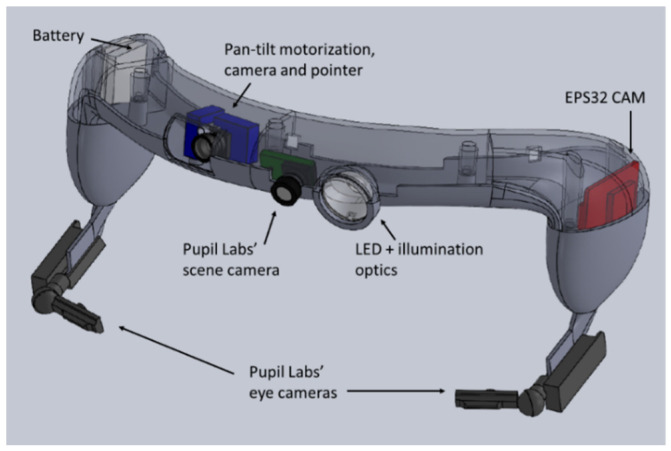
CAD view of the designed headband illustrating the position of the different integrated elements: the two eye tracker’s cameras in front of the eyes and the scene camera, the ESP32 and batteries placed on the sides, and the pan tilt supporting the motorized camera and the pointer.

**Figure 3:**
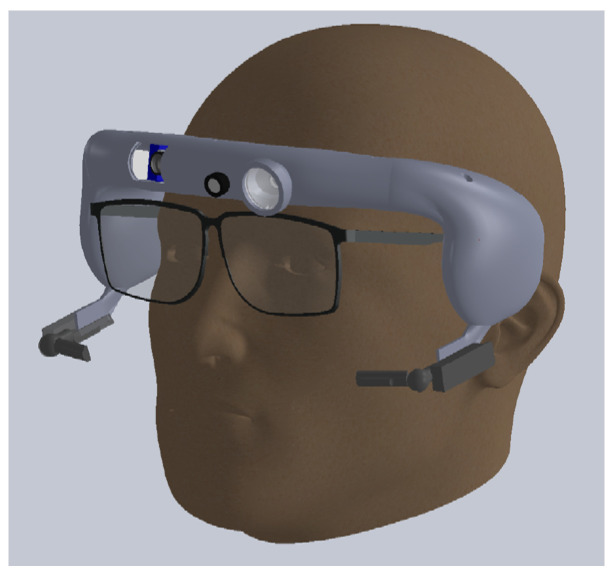
Illustration of the headband on a face. The headband was designed so that it could be used by spectacle wearers. We can distinguish the two cameras of the eye tracking module which are under the eyes, slightly in front of the glasses.

The headband system integrating the various components was designed using the SolidWorks software to be manufactured by additive fabrication. The advantage of this solution is to allow rapid prototyping in order to be able to adapt to the expectations of end users (e.g., surgeons). The specific headband layout and the different integrated elements are represented in Figure 2 and Figure 3. The headband weights 111 g and the material used is Phrozen Water Washable Rapid Black Resin. A specific design was chosen to place the cameras near the eyes for a “first-person” viewpoint and to allow users to wear spectacles or binoculars.

### 2.6. Electronic Control

In addition to the motorization, the control unit is obviously a key element to manage the data stream towards the user. The electronic device used here to control all the components but the eye tracker was an ESP32-CAM microcontroller (model CPU 240 MHz, RAM: 520 Ko, Wi-Fi: 802.11 b/g/n) that comes with a SD card slot and ribbon cable interface for a camera. It is a relatively small (27 × 40 × 4.5 mm, 10 g), low-cost (~$5), low-power system with integrated Wi-Fi and dual-mode Bluetooth. To be easily operated on an embedded and autonomous headband, the camera, motorization, micro-controller and light sources were powered by two batteries: Li-ion Polymer (3.7 V 130 mAh 7.7 g each), placed on the sides of the headband as depicted in Figure 2. Alternatively, larger batteries could be attached to the headband with a strap (total weight 166 g) and placed behind the head (occipital region) rather than on the headband for improved comfort.

### 2.7. System Operation

**Figure 4:**
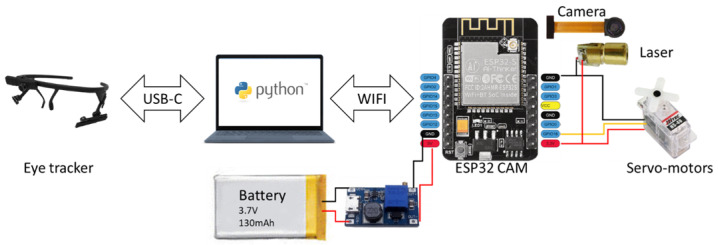
Diagram of the control and communication channels of the various elements of the headband. The electronics between the battery and ESP32 is used to step-up the voltage to 5 V.

The camera output is both recorded locally and transmitted wirelessly. The control and communication channels of the various modules making up the system are depicted in Figure 4. In this case, the eye tracker is driven by software installed on a laptop computer, connected to it by a USB-C cable for the transfer of the scenes captured by the two cameras before real time analysis. A Python program retrieves the eye tracker data, calculates the correct angular command (pan and tilt pulse-width modulation (PWM) values) for the servos and sends it via Wi-Fi channel to the ESP32 that controls the servos. At the same time, the ESP32 retrieves the video from the camera and connects to an existing local Wi-Fi network from which it gets an IP address. Using this information, it also sets up a server that another Python script can use to download and display the video.

In the next version, we shall integrate a fully embedded system for which these operations are entrusted to an autonomous micro-computer (e.g., Raspberry Pi), which could also manage the servo-motors and the piloting of the laser-pointing sources.

## 3. Results

### 3.1. Integration

**Figure 5:**
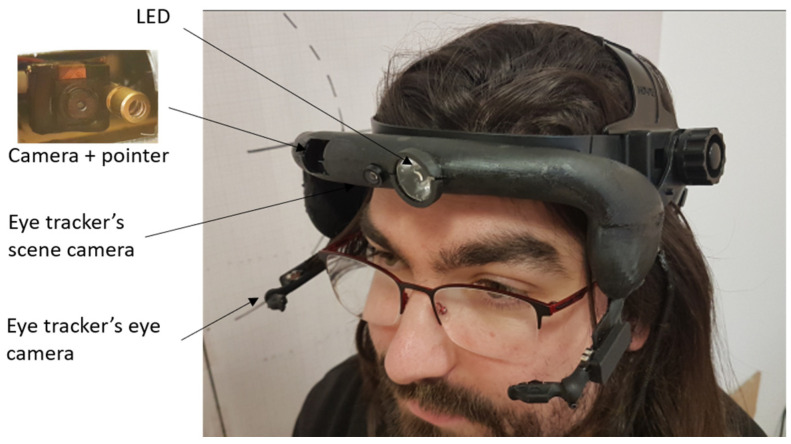
3D-printed headband following the model presented in Figure 2 and integrating the different components. The rigid headband mount can be easily replaced by a headband strap.

The different elements presented in Section 2 were successfully housed into a specifically designed ergonomic headband that overall weighs 396 g (incl. eye tracker, batteries, etc.). The device worn by a human operator is depicted in Figure 5. Despite its slight lack of flexibility regarding the possible on-demand switching of sources, the laser diode option was chosen over the fibered laser because the small volume available for integration imposes on the fiber strong radii of curvature, which weakens it.

### 3.2. Pointing Precision

**Figure 6:**
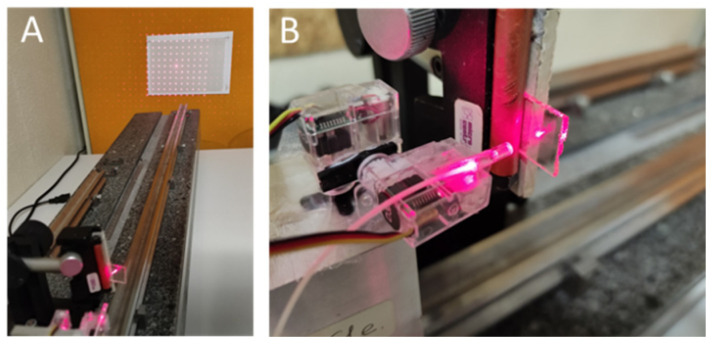
(**A**): test bench. (**B**): the fibered laser is on the pan/tilt motorization and illuminates the glass plate where is the optical diffractive element.

The laser-pointing module was used to test the precision of the angular control allowed by the pan/tilt motorization. The system was set on an optical test bench and pointed at a piece of graph paper 85 cm away, as depicted in Figure 6. The first angular actuator tested was the one using the nano D1302 servo-motor.

**Figure 7:**
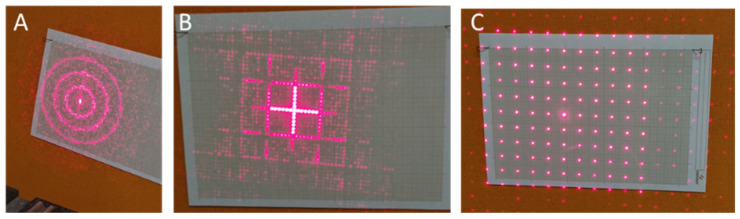
Examples of light distribution patterns obtained with different DOEs: circular target (**A**), cross-hair (**B**), regular dot matrix (**C**).

The deadband was assessed by first increasing the pulse-width modulation (PWM) in steps of 1 μs, starting from an arbitrary value, until motor activation then by repeating the process but in decreasing PWM values until no motor reaction. The process was repeated multiple times to confirm a servo-motor deadband value of 25 μs. To evaluate the accuracy and precision of the pointing system, a 50 × 50° square field was considered. On this area, five reference points were considered, on which the beam was pointed repeatedly. These points were chosen at the center and at the four corners of the square area. For each test, a measurement of the actually addressed position was made and compared to the target position. A total of 250 measurements were made in random order, i.e., 50 measurements per point. The horizontal field tested is smaller than the camera’s field of view, but in practice our eyes do not rotate more than 16°, after which we turn our heads instead. A moderately good value of 1.3° was obtained for the tracking precision, mainly due to the non-zero driving electrical deadband value. Unfortunately, this factory setting cannot be changed on this type of miniature component. Moving to the HS-40, the tracking precision was improved to 0.15 degrees. The angular repeatability was, however, limited to 0.35°, again due to the non-zero value of the servo deadband (4 μs), resulting in a maximum spot positioning error of 5 mm at 80 cm. Note that the impact of this value on the overall accuracy of the system is minimal, as the Pupil Labs eye-tracker accuracy is 0.6°. Examples of spatial light distribution patterns obtained with different DOEs are also depicted in Figure 7, in the projection area located 80 cm from the headband.

When tested with the eye tracker, the latency with which the motorized camera followed the gaze direction was assessed by recording a video in which the subject wearing the device directed his gaze at different targets while pointing at them. Despite the high servo-motor speed, the latency was approximately 0.5 s due to data processing and data transmission. A simple video illustrating the use of the device for remote assistance is available as Supplementary Material.

### 3.3. User Feedback

**Figure 8:**
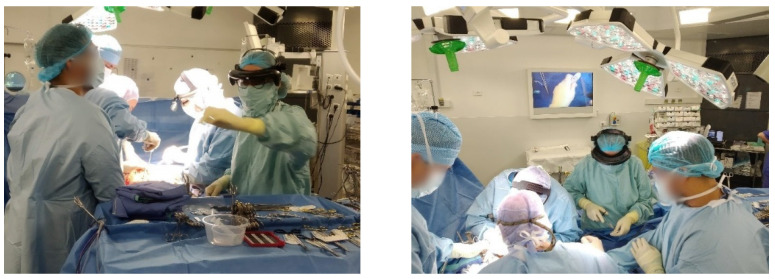
Operating theater nurse wearing the headband. Left image: streaming from the camera is displayed on the wall monitor.

The system was presented to and used by surgeons from Paul Brousse Hepato-Biliary Centre in Paris. The feedback on design and integration was globally positive. A nurse from the surgical unit in charge of the logistics of the operating theater wore it for 90 min (Figure 8) and confirmed the good ergonomics (i.e., it was comfortable to wear and did not disrupt her activity). The limited image quality of the live video (400 × 400 pixels) was, however, perceived as a hindrance to the immersion of outside observers in the scene visualized by the holder of the device. Recall that the widespread micro-camera model used here allows recording high-quality images on the local SD card (1600 × 1200) but is not adapted for real-time streaming of high-quality videos.

### 3.4. Improved Optical Pointing

**Figure 9:**
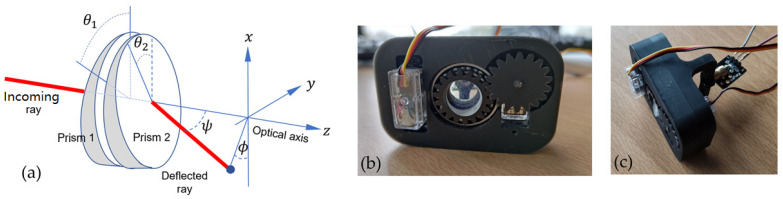
(**a**) Risley-prism-based deflection system with output ray ϕ azimuth and ψ elevation angles. (**b**) Mini-prisms and angular mount driven by servo-motors (front view). (**c**) Top view of the integrated deflection optical head unit with input laser source.

As previously stated, fluid and precise motorization is essential for the device to be fully functional. In view of the limited performance obtained with the pan-tilt motorization, an alternative solution based on Risley prisms was designed and tested. Risley prisms (RPs) are widely used beam-deflection mechanisms due to their high accuracy, low moment of inertia, and low cost [18]. They are used as beam-steering and scanning devices in inter-satellite and airborne laser communications [19], wide field-of-view imaging, lidar, guidance systems and wavefront alignment and positioning. A RP system consists of a pair of optical wedges that rotate independently about a common axis (Figure 9a), allowing arbitrary beam pointing with high resolution and wide angular range. The elevation (Ψ) of the emerging beam is controlled by the difference (θ_1_ − θ_2_) between the rotation angles of the two prisms, while the azimuthal angle (Φ) is modified by a synchronized co-rotation of the pair.

The main advantage of the device is the simplicity of control: the scanning is purely mechanical and only concerns the rotation of the prisms. In addition, the movement is mechanically balanced (low moment of inertia), unlike the pan-tilt mount presented in the previous sections. In most of the above-mentioned applications, the RP devices are large and heavy (several kilograms) due to the use of massive metal mounts coupled with bulky DC or steeper motors. In the present case, we have designed a miniature RP-based deflector, whose objective is to show that the size of such a device can be reduced to a scale comparable to that of our headband so that it can be integrated into it. To this aim we first select circular mini-prisms with 10 mm diameter and an apex angle of 15°. They are mounted in gear wheels (Figure 9b) driven by mini servo-motors (mass 15 g) with angular range and angular resolution of 270° and 0.1°, respectively. The scanning speed of the servo-motors is 500°/s. The prototype optical head was manufactured by 3D printing. Its dimensions are 5 × 7 × 3 cm and its mass is 150 g. It is equipped as standard with a 2 mW laser micro-pointer. Again, the angular motion control system is managed by an ESP32 microcontroller, which sends two PWMs signals to the servo-motors to control the relative angles of the prisms. At the end of an experimental test phase similar to that of the pan-tilt-based optical head, the RP option has proved to be twice as accurate and faster than the pan-tilt system, with a more “fluid” and repeatable dynamic angular positioning of the laser spot, due to a better mechanical balance of the mobile structure. This solution, therefore, represents an improvement in results, and the current step is to integrate the RP-based optical head (Figure 9c) into the headband. This is not a major obstacle with the design and 3D-printing techniques employed here.

## 4. Discussion

The ability to record a scene as seen by the observer and to materialize the location where his visual attention is projected, as well as the ability for an external observer to point to an element of the observed scene, could be beneficial for a number of applications such as surgery. In this context, we presented a light headband (396 g with batteries) that integrates a motorized camera and a laser pointer that can be remotely controlled or driven by eye movements. We have also demonstrated the use of a custom DOE to shape the laser beam into structured patterns, if required by the application.

In terms of manufacturing and integration, the use of 3D printing to produce the prototype made it possible to integrate off-the-shelf components without particular difficulty and to test various designs. We chose here a head-mounted device, as we focused on its application in surgery, but the same components (albeit without the the eye tracker) could be easily integrated in another design, e.g., a body-worn camera as part of policing equipment.

In terms of performances, hobby servo-motors are performant enough to control the pan-tilt motorization with satisfactory accuracy, though the servo-motor deadband leads to limited performance when rapid and accurate control is needed (eye tracking case). Similarly, small, inexpensive lasers and cameras can provide efficient pointing or structured illumination (in conjunction with a DOE) and high-quality images.

In the presented configuration, the camera could be rotated over ±20° in both direction. This angle is defined by the size of the opening in the headband, since beyond this angle, the camera’s field of view will be blocked by the edges of the headband shell. The impact in relation to the chosen camera. which had a large field of view. was limited, but it could easily be replaced by a zoom camera to complement the scene camera from the eye tracker. The resolution, sensitivity and field of view of a camera may not always be appropriate for all imaging conditions, and a second camera could help address this issue. It could even be used to provide 3D information which could be valuable (e.g., for tele-expertise).

In addition, we proposed two alternatives for the dynamic optical head technology: (i) one, directly integrated in the headband, based on a pan-tilt movement directly transmitted by mini-servo motors, but limited in precision and angular reactivity, and the other, (ii) more performant and based on Risley prisms, requiring an additional mechanical integration effort. As well as being dependent on the angular performance of the optical beam-steering module, our system is mainly limited by two issues: the latency with which the camera follows the gaze direction and the quality of the live streaming. The ESP32 has difficulty managing the different information streams simultaneously. As a result, the system cannot effectively stream high-resolution images (i.e., 1080p) and the motorized camera movement can be delayed by half a second. These two points are likely to limit the interest of the eye tracker to control the camera or pointer.

Embedding some computing power into the headband (e.g., the Pupil Labs software can run on a Raspberry Pi) would allow for a fully wearable device but would not significantly reduce the ESP load. For instance, a Raspberry 4 (ARM quad-core, 1.8 Ghz) allows for recording the images of both eyes at a stable frequency of 124 Hz for post-treatment, but only allows eye tracking at a maximum 80 Hz or 40 Hz, depending if two or one eye is considered (versu 120 Hz/90 Hz if images are transmitted by Wi-Fi). We are currently studying a solution based on a micro-controller with increased computing capabilities, but without increasing the cost, weight, compactness and electricity consumption.

However, high-resolution video streaming may not be necessary for a number of applications. The high-quality recording on the SD card is enough for documenting, and activities such as monitoring or surveillance do not need high-resolution images. For such applications, an interesting alternative could be to switch to analog video transmission so as to benefit from the available flight controller card developed for drones and radio control hobbyist.

Another source of improvement consists of using a contact lens eye tracker, as we are developing in parallel [20,21], to simplify gaze detection and improve the headband functionality.

In addition to the applications cited earlier, one of interest would be vision substitution [22]. Substitution strategy aims at informing a visually impaired subject about their surrounding environment by providing them with relevant details through audio or tactile channels. For instance, the user scans the environment with a camera placed on their head and receives information about obstacles. In this context, a camera controlled by eyes rather than head movements could be much more comfortable for the user and provide more relevant information (assuming a reasonable control of eye movements).

## Data Availability

All relevant data is presented in this manuscript.

## Supplementary Materials

A video (VID1) is available at https://doi.org/10.17605/OSF.IO/UQVF6, accessed on 17 March 2023.
